# Supplementary material for: CD10-Bound Human Mesenchymal Stem/Stromal Cell-Derived Small Extracellular Vesicles Possess Immunomodulatory Cargo and Maintain Cartilage Homeostasis under Inflammatory Conditions
Source: Cells. 2023 Jul 11;12(14):1824. doi: 10.3390/cells12141824 (PMC10377825; doi:10.3390/cells12141824)
Supplement: Supplementary file 1 [file cells-12-01824-s001.zip › Supplementary Table S1.pdf]

Supplementary Table S1. Human mesenchymal stem cell qPCR array plate genes and their classification.

**TRANSCRIPT NAME**	**CLASSIFICATION**
PPARG, RHOA, CEBPA, CEBPB, LEPR	Adipogenic
HAT1, ITGAX, KAT2B, SOX9, ACAN, COL2A1, MMP13, CSPG4	Chondrogenic
BMP4, TGFB1, TGFB3, BMP6, KDR, MSX1, MSX2, BMP2, PRRX1	Chondrogenic / Osteogenic
TWIST2, ANPEP, CASP3, CD44, ENG, ERBB2, FUT4, FZD9, ITGA6, ITGAV, MCAM, NGFR, NT5E, PDGFRB, PROM1, THY1, VCAM1, BDNF, CD200, COL10A1, FGF18, DLX2, DLX5, PDGFRA	MSC
CTNNB1, EGF, HGF, ICAM1, IFNG, IGF1, IL10, IL1B, IL6, ITGB1, KITLG, MMP2, NES, NUDT6, PTPRC, SLC17A5, TNF, VEGFA, VIM, VWF, JAG1, NOTCH1, GDF15, SMAD4, HIC1	MSC-related/Angiogenesis
ALPL, IBSP, SP7, BGLAP, BMP7, COL1A1, FGF10, HDAC1, PTK2, SMURF1, SMURF2, TBX5, RUNX2, FGF9	Osteogenic
FGF2, LIF, SOX2, TERT, GDF5	Stemness
GAPDH, RN18S1, ACTB, TBP, UBC<br>Positive Control	Housekeeping / Control
