# Supplementary material for: CD10-Bound Human Mesenchymal Stem/Stromal Cell-Derived Small Extracellular Vesicles Possess Immunomodulatory Cargo and Maintain Cartilage Homeostasis under Inflammatory Conditions
Source: Cells. 2023 Jul 11;12(14):1824. doi: 10.3390/cells12141824 (PMC10377825; doi:10.3390/cells12141824)

Supplementary Table S2. The colors for different evidence codes and for log scale in the g:Profiler functional enrichment analysis.

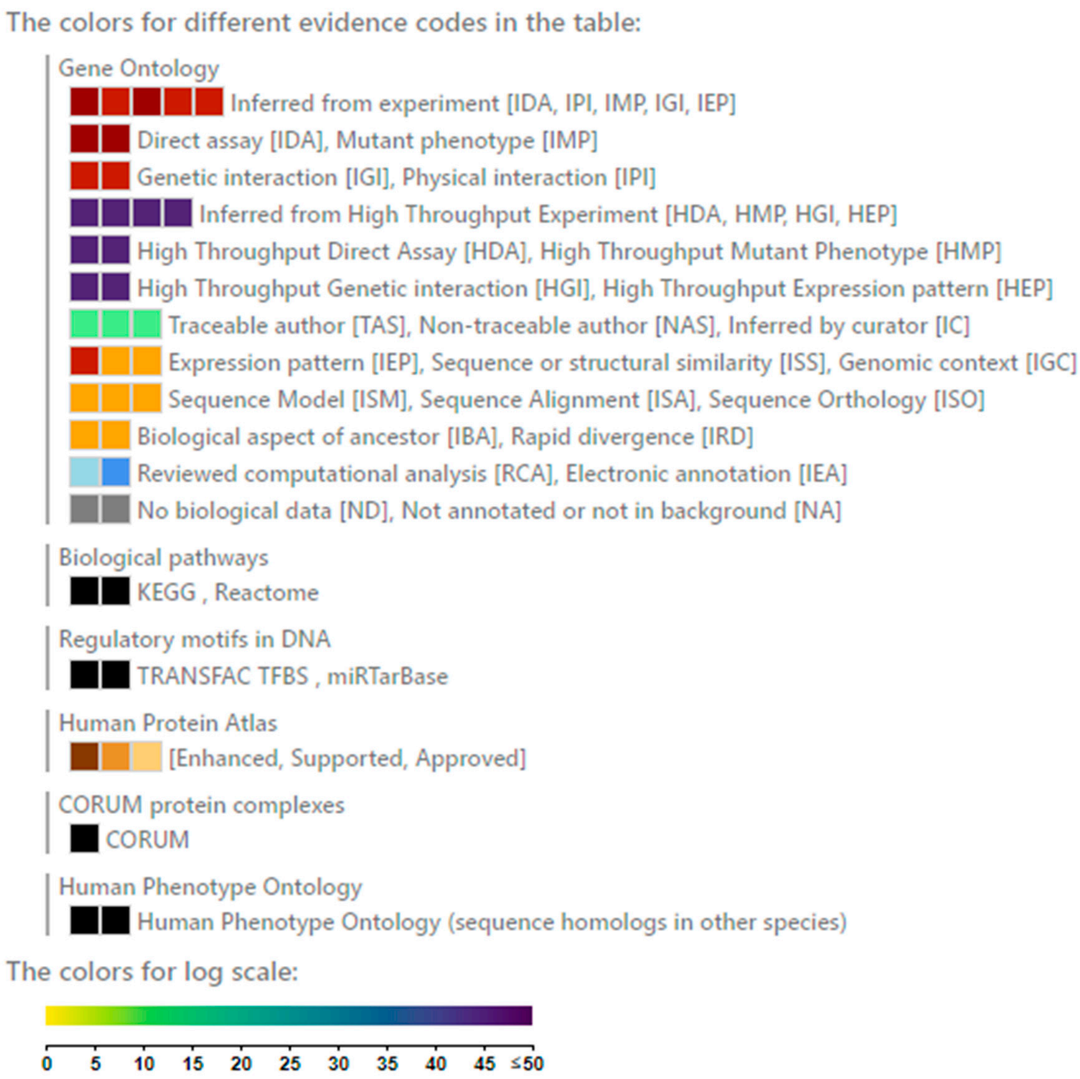

Supplement: Supplementary file 1 [file cells-12-01824-s001.zip › Supplementary Table S2.pdf]
